# Behavior therapy for pediatric trichotillomania: Exploring the effects of age on treatment outcome

**DOI:** 10.1186/1753-2000-4-18

**Published:** 2010-06-28

**Authors:** Martin E Franklin, Aubrey L Edson, Jennifer B Freeman

**Affiliations:** 1 Department of Psychiatry, University of Pennsylvania School of Medicine, Philadelphia, Pennsylvania, USA; 2 Department of Psychiatry, Alpert Medical School of Brown University, Providence, Rhode Island, USA

## Abstract

**Background:**

A randomized controlled trial examining the efficacy of behavior therapy for pediatric trichotillomania was recently completed with 24 participants ranging in age from 7 - 17. The broad age range raised a question about whether young children, older children, and adolescents would respond similarly to intervention. In particular, it is unclear whether the younger children have the cognitive capacity to understand concepts like "urges" and whether they are able to introspect enough to be able to benefit from awareness training, which is a key aspect of behavior therapy for trichotillomania.

**Methods:**

Participants were randomly assigned to receive either behavior therapy (N = 12) or minimal attention control (N = 12), which was included to control for repeated assessments and the passage of time. Primary outcome measures were the independent evaluator-rated NIMH-Trichotillomania Severity Scale, a semi-structured interview often used in trichotillomania treatment trials, and a post-treatment clinical global impression improvement rating (CGI-I).

**Results:**

The correlation between age and change in symptom severity for all patients treated in the trial was small and not statistically significant. A 2 (group: behavioral therapy, minimal attention control) × 2 (time: week 0, 8) × 2 (children < 9 yrs., children > 10) ANOVA with independent evaluator-rated symptom severity scores as the continuous dependent variable also detected no main effects for age or for any interactions involving age. In light of the small sample size, the mean symptom severity scores at weeks 0 and 8 for younger and older patients randomized to behavioral therapy were also plotted. Visual inspection of these data indicated that although the groups appeared to have started at similar levels of severity for children ≤ 9 vs. children ≥ 10; the week 8 data show that the three younger children did at least as well as if not slightly better than the nine older children and adolescents.

**Conclusions:**

Behavior therapy for pediatric trichotillomania appears to be efficacious even in young children. The developmental and clinical implications of these findings will be discussed.

**Trial Registration:**

Clinicaltrials.gov NCT00043563.

## Background

Trichotillomania (TTM) is a chronic impulse-control disorder in which the individual pulls out one's hair to the point of alopecia. TTM is estimated to affect 1% - 3.5% of late adolescents and young adults [1]; rates among younger children are largely unknown [2]. Sufferers of TTM across the developmental spectrum may experience medical complications such as skin irritation, infections and repetitive use hand injuries [3]; those who ingest the hairs after pulling are at risk for gastrointestinal complications stemming from trichobezoars (i.e., hairballs); [4,5]), which have been documented in patients as young as four [6]. Psychiatric comorbidity is apparently common, and includes anxiety disorders, mood disorders, substance use disorders, eating disorders [6,7], and personality disorders in adults [8] and anxiety and disruptive behavior disorders in youth [9,2]. Notably, TTM onset in childhood or adolescence appears to be the norm, and TTM onset typically precedes that of most comorbidities [10]. Accordingly, a major priority in TTM psychopathology and treatment research is to recruit younger samples since clinical trials have enrolled children with TTM as young as seven, so we know that they can participate, if not do well, with Habit and Reversal Training (HRT) [11]. The goal of improving our understanding of TTM closer in time to its onset will perhaps, by extension, reduce future functional impairment and prevent the development of debilitating comorbid disorders.

Despite the fact that TTM is a pediatric onset disorder associated with significant morbidity, comorbidity, and functional impairment in adults [12], very few TTM psychopathology research studies have actually included adolescents or children, and there are as yet no published randomized controlled trials (RCTs) of any psychopharmacological interventions for youth with TTM. Initial findings for cognitive-behavioral therapy were encouraging [13], but questions pertaining to the role of developmental factors in TTM psychopathology and treatment response have yet to be examined. With respect to similarities and differences in TTM presentation across development, very little is known about symptom presentation in young children, but it appears that the scalp is the most common pulling site in both adults and older children and adolescents [7,14,2,15]. Pulling tends to be both automatic (i.e., outside awareness) and focused (i.e., in response to identifiable affective triggers) within each individual, rather than exclusively one form or the other [16,17] although it appears that there is a greater preponderance of automatic pulling in younger samples. The concept of urge plays an important role, as most participants in TTM studies to date have reported tension or some other unpleasant sensation that precedes pulling [12]. Whether urges are present or can be reliably described by younger patients with TTM is unknown, although one study among youth and adults with tic disorders [18] found that while adults were able to identify and verbalize both the premonitory urge to tic and the relief experienced after indulging that impulse, children under age 10 were unable to describe the premonitory urge reliably. Perhaps young children have not yet developed the expressiveness skills and emotional awareness [19] required in behavior therapy for TTM, so it is unclear if young children would actually benefit from such treatments. Further, as Freeman et al.'s [19] research demonstrated among young children with OCD, very young participants may lack the insight, motivation, and developmental capacity to follow a treatment protocol on their own, so the protocol may need to be altered to better suit these developmental needs and to set the treatment in the context of the family.

Following from Freeman et al.'s [19] work, we wish to examine whether the developmental issues described above necessarily preclude the use of child-focused HRT in the treatment of young children with TTM. For the reasons outlined above, we believe that HRT designed for older children and adolescents with TTM will yield the same gains in treatment outcome when applied to young children. Further, given that there are no prior published randomized trials for any treatment for pediatric TTM and that there is a paucity of information available about TTM psychopathology and treatment outcome in younger children with TTM, data in the current report are being used solely for the purposes of hypothesis generation.

## Methods

### Participants

Participants were recruited into a randomized controlled trial examining the efficacy of behavior therapy for pediatric TTM that was conducted at the University of Pennsylvania's School of Medicine. Primary inclusion criteria for that study were: 1) ages 7 -17 inclusive; 2) diagnosis of TTM; 3) symptom duration of at least six months; and 4) participant and at least one parent fluent in English. Primary exclusion criteria were: 1) a primary psychiatric diagnosis other than TTM; 2) current bipolar illness, developmental disorder, or thought disorder; and 3) currently receiving either pharmacotherapy or concomitant psychotherapy for TTM. Written informed consent was obtained from all participants for publication of this manuscript and accompanying images. A copy of the written consent is available for review by the Editor-in-Chief of this journal. Twenty-four patients were randomized, 12 to each condition. The sample ranged in age from 7 - 17, with a mean age of 12.5 (2.7); 4 of the 24 participants (17%) were ≤ 9 years old, with 3 of those participants randomized to BT. The sample was primarily Caucasian (75%), primarily female (67%), and had a mean age of TTM onset of 8.9 years (3.2). Additional information about participants randomized to BT can be found in Table 1. Notably, all 24 patients completed the acute phase (week 8) of treatment, and all 12 patients randomized to BT completed the 8-week maintenance phase. Because all MAC patients were offered open BT at week 8 on ethical grounds, no data on MAC are available through the maintenance phase of the study. Only data from the acute phase of the study will be presented in the current report.

**Table 1:** Duration of Pulling, Age of Onset and Number with Comorbidity in Those Assigned to Behavioral Therapy

	Average Duration of Pulling (years)	Average Age of Onset (years)	Percentage with Comorbidity
**Children ≤ 9 **(*N *= 3)	3.0 (SD = 2.6)	5.3 (SD = 2.1)	33%
**Children > 9 **(*N *= 9)	4.0 (SD = 2.3)	8.4 (SD = 3.9)	33%

### Measures

Diagnostic criteria for TTM were assessed using the Trichotillomania Diagnostic Interview (TDI) [20], which examined TTM diagnosis according to DSM-IV criteria; notably, as in other studies of TTM, Criterion B (tension prior to pulling) and Criterion C (gratification/relief following pulling) were evaluated yet not required for study entry. Diagnostic criteria for other psychiatric disorders were surveyed using the Anxiety Disorders Interview Schedule for Children (ADIS-C) [21], a semi-structured interview with established psychometric properties. The TDI and ADIS-C were used to assess diagnostic inclusion criteria, and were conducted at intake by evaluators trained to criteria in their use. The primary measure of treatment outcome was the NIMH Trichotillomania Severity Scale (NIMH-TSS) [22], which has demonstrated adequate psychometric properties in prior studies of TTM treatment [see [23]]. The NIMH-TSS ranges from 0 (no symptoms) to 25 (severe symptoms), and surveys time spent pulling in the past week, time spent pulling the previous day, resistance to pulling, associated distress, and functional impairment. Trained independent evaluators blind to treatment assignment conducted the NIMH-TSS interviews at weeks 0, 4, and 8 during the acute phase of the trial; the same evaluator conducted assessments throughout the trial to minimize interviewer effects. A Clinical Global Impression - Improvement (CGI-I) score was also rated at week 8 by the same blind evaluator; this scale ranged from 1 (much worse) to 7 (much better), with a score of 4 indicating no change in symptoms from baseline. Only data from weeks 0 (baseline) and 8 (post-treatment) assessments are presented in the current report.

### Treatments

Behavior therapy was conducted in accordance with a manual developed in the context of a treatment development grant; this manual has now been published [24]. The acute treatment phase for behavior therapy lasted eight weeks and was conducted weekly; core elements of treatment included: 1) psychoeducation about the nature and treatment of TTM; 2) awareness training, in which participants were taught to become more aware of pulling behavior and pulling urges; 3) stimulus control, in which barriers to pulling were created based on participants' report of high-risk situations; and 4) competing response training, in which participants were taught to engage in behaviors that were physically incompatible with pulling. Ancillary strategies were also permitted and included: progressive muscle relaxation (Session 5) and cognitive restructuring (Session 6); inclusion of these strategies was discussed in weekly supervision meetings with the Principal Investigator (MEF). Minimal attention control (MAC) was employed in this treatment development project to control for the effects of time and of repeated assessment; participants who received MAC were introduced to a therapist at week 0 and met again with the therapist at weeks 4 and 8. Notably, MAC did not match BT in the amount of clinical contact, nor was it intended to be an active intervention. Accordingly, MAC participants were offered open BT at week 8, thus no comparisons of BT and MAC were possible beyond week 8. Primary outcomes for the comparison of BT versus MAC are presented elsewhere [11] but indicated a clear advantage for BT over MAC following acute treatment.

### Statistical Methods

Study data were examined using three main approaches. First, the correlation between participants' age and change in TTM symptoms over the course of treatment was calculated. Second, combined plots were created to permit visual inspection of response trends between the older (≥ 10) and younger (≤ 9) participants; this dichotomy was selected based on data from Tourette Syndrome indicating that children ages 9 and lower have more difficulty reliably reporting on concepts such as urges [see [18]]. Along with this standard, single-subject approach to data analysis, an exploratory, mixed repeated-measures analysis of variance (ANOVA) was conducted to test for differences between the older and younger groups in IE-rated TTM severity over time. The NIMH-TSS pre-treatment distribution of scores appeared to be normal but, as is often the case in clinical trials in which the treatments are active, there was some evidence of negative skew in the distribution at post-treatment; nevertheless, ANOVAs were conducted to help better contextualize study findings against the broader literature on TTM. Notably, the current study was not powered to conduct traditional significance testing across multiple dependent measures, hence the exclusive focus on the study's primary continuous outcome measure, the blind IE-rated NIMH TSS scores.

## Results and Discussion

### Correlation between Age and Change in TTM Symptoms

The correlation between age and change in TTM symptoms (NIMH-TSS total score at week 0 - week 8) for all 24 participants in the RCT was -.16, which was not significant statistically (*p *= .48) and not supportive of an expected association between age and change in TTM symptom severity over time regardless of treatment received.

### Group by Time Effects

A 2 (condition: BT, MAC) × 2 (time: week 0, 8) × 2 (age group: children ≤ 9 yrs., children ≥ 10) ANOVA was conducted, with IE-rated NIMH-TSS scores as the dependent variable. No main effect for age was detected, nor did any interactions involving age emerge (all Fs < 1.0). Nevertheless, because children ≤ 9 comprised only 17% of the sample, statistical power to detect differences is inherently limited. Although statistical comparisons were not conducted, data on percentages of those with improved or very much improved CGI-I scores are presented in Table 2.

**Table 2:** Pre and Post Treatment NIMH-TSS Scores, NIMH-TSS Effect Sizes and Percentage Improved or Very Improved on the CGI-I

	Pre-Treatment NIMH-TSS Scores *(M)*	Post-Treatment NIMH-TSS Scores *(M)*	NIMH-TSS Within-Subjects Effects Size (Cohen's d)	Percentage Improved or Very Much Improved on the CGI
**Children ≤ 9 ***(N = 3)*	11.2 (SD = 2.3)	0.7 (SD = 1.9)	2.68	100%
**Children **>**9 ***(N = 9)*	12.7 (SD = 3.0)	4.4 (SD = 1.4)	1.19	67%

### Visual Inspection of Plotted NIMH-TSS Data

**Figure 1:**
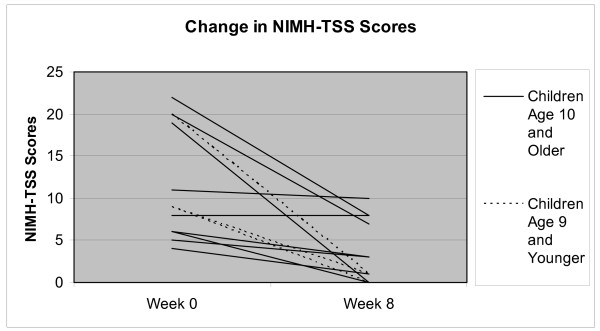
**Change in NIMH-TSS Scores**. This figure represents the change in NIMH-TSS scores from week 0 to week 8 for each of those participants randomized to BT

**Figure 2:**
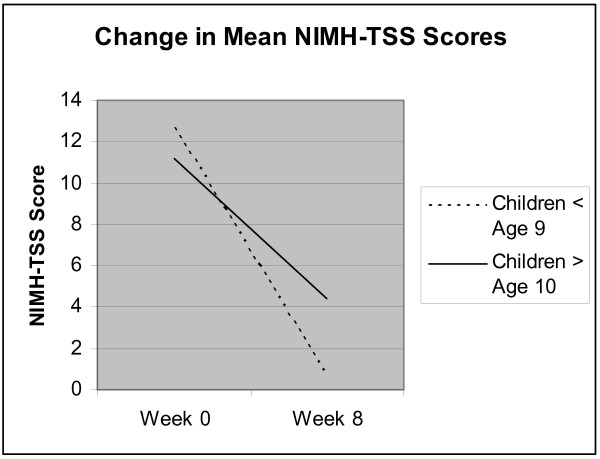
**Change in Mean NIMH-TSS Scores**. This figure represents the mean change in NIMH-TSS scores from week 0 to week 8 for those participants randomized to BT; the figure represents the difference between mean changes among the participants who were age 9 or younger and participants were age 10 or older.

In order to further explore the possible influence of developmental factors on behavior therapy outcomes specifically, all NIMH-TSS scores at weeks 0 and 8 for younger and older patients randomized to BT were plotted and visually inspected as evident in Additional File 1 (Figure 1), and the mean NIMH-TSS scores at pre and post-treatment for both age groups are presented in Additional File 2 (Figure 2). Although, NIMH-TSS scores at week 0 appear to be quite similar ((M = 12.7 (4.0) for children ≤ 9 vs. M = 11.2 (2.3) for children ≥ 10)) and the within-subjects effect sizes for both groups were very large (see Table 2), the week 8 data suggests that the three younger children ((M = 0.7 (1.9)) did at least as well as if not slightly better than the nine older children and adolescents ((M = 4.4, (1.4)), although developmental issues such as ability to understand evaluators' questions about urges and recall pulling behavior over the course of the past week may decrease confidence in the outcomes for the young children.

## Conclusions

The purpose of the current report was to explore whether developmental factors influenced change in TTM symptoms in a pediatric sample randomly assigned to receive either behavior therapy or a comparison condition designed to control for the effects of time and repeated assessments. Findings indicated that there was a small, negative, and insignificant relationship between participants' age and change in TTM symptoms over the course of the acute phase of the trial. When a mixed ANOVA was employed to further explore the effects of age on treatment outcome, here again no effects for developmental level emerged. Low power could have obscured such effects, however, so inspection of behavior therapy outcomes specifically was warranted in order to generate hypotheses about whether the youngest participants might have experienced attenuated outcomes relative to their older counterparts. The behavior therapy protocol for TTM includes techniques that require at least some ability to introspect (e.g., awareness training), and the tailoring of subsequent treatment strategies such as stimulus control and competing response training rest upon increased awareness of the presence of urges to pull. Our data here were somewhat surprising given initial concerns about the ability of young children to grasp these core concepts in that their outcomes were clearly not attenuated, and were even suggestive of the possibility that children age 7 - 9 might experience more success in BT than their older counterparts.

Data emerging from cross-sectional comparisons of TTM in different developmental stages might help explain this seemingly anomalous observation, in that it appears that pulling in younger children might be more "automatic" and less affect-driven than it appears to be in older children and in adults [16]. Younger children also report fewer pulling sites than do older children and adults [25], again suggesting that TTM might become more complex over time. Driven by accumulating evidence that relapse is common following BT in adults [26], current treatment development research in adult TTM has focused recently on incorporating treatment components to address emotions specifically [27]. What cannot be examined using data from the current study, however, is whether developmental factors such as inability to introspect or report on concepts such as urges would negatively impact treatment of children younger than age 7. There is now evidence in pediatric OCD indicating that a family-based intervention involving exposure plus response prevention for children ages 4 -8 was superior to a relaxation control condition [19]; it may well be the case that similar treatment development efforts in pediatric TTM will permit effective intervention that can be delivered to children even younger than those who participated in the current study. Our findings of comparable if not slightly better treatment outcomes for younger children are not entirely consistent across pediatric onset disorders in which urges play a prominent role such as in Tourette Syndrome; thus, cross-diagnostic studies are clearly needed to examine urge phenomenology and response to behavioral treatments such as HRT in which urge awareness may play a prominent role in signaling the patient to engage in a competing behavior. Such efforts are indeed underway, and will closely mirror efforts already being made in OCD specifically to properly contextualize the behavioral intervention in view of developmental considerations, given the misinformation about these conditions that many families have been exposed to prior to seeking treatment, and the deleterious interactive effects of these conditions on family environment and on specific family members.

All of the limitations inherent in any study with a sample size this small are applicable here as well, and thus even our preliminary conclusions must be interpreted with considerable caution. Nevertheless, the dearth of published research in pediatric TTM necessitates efforts at empirical hypothesis generation wherever possible, which is what led to the exploration reported above. A second randomized controlled trial funded by the National Institute of Mental Health in the U.S. that compares the efficacy of BT to a more active control condition that equates therapist contact time (Psychoeducation/Supportive Counseling) is underway at Penn but, unfortunately, reviewer concerns about the developmental issues discussed above necessitated a design decision to truncate the age range to 10 - 17. There are very few other sources of knowledge available about treatment efficacy for children with TTM who are younger than age 10, and thus we felt that, despite the obvious caveats, it was important to take the opportunity provided by these data to help stimulate thinking about whether and how best to intervene in young children with TTM.

## Competing interests

The authors declare that they have no competing interests.

## Authors' contributions

ALE wrote the Backgrounds section and was responsible for formatting responsibilities. MEF was the study investigator and authored the majority of the manuscript. MEF and JBF conducted analyses, interpreted the data and revised the manuscript. ALE, MEF and JBF reviewed and approved the final version of the manuscript.
